# Evaluating flourishing: a comparative analysis of four measures using item pool visualization

**DOI:** 10.3389/fpsyg.2024.1458946

**Published:** 2024-11-06

**Authors:** Veronika Ploke, Bernad Batinic, Stefan Stieger

**Affiliations:** ^1^Department of Psychology and Psychodynamics, Karl Landsteiner University of Health Sciences, Krems an der Donau, Austria; ^2^Department of Work, Organizational and Media Psychology, Johannes Kepler University Linz, Linz, Austria

**Keywords:** flourishing, wellbeing, positive psychology, item pool visualization, structural equation modeling, self-report

## Abstract

The construct of flourishing, which refers to a high level of wellbeing, is a key concept in the field of positive psychology. Nevertheless, despite the proliferation of questionnaires attempting to measure wellbeing and flourishing, there is still an ongoing debate in the scientific community about the definition and assessment of both, which makes the choice of appropriate measures a major challenge. This study addresses this uncertainty through a comparative analysis of four widely used flourishing measures in a German-speaking sample, to enable researchers to make a reasonable choice among the measures available: the PERMA-Profiler, the Mental Health Continuum-Short Form, the Flourishing Scale, and the Wellbeing Conceptual Framework. To enhance the understanding of these four measures, we compared and contrasted the instruments using Item Pool Visualization (IPV), an illustrative approach that generates item pools from the same dataset and displays them as nested radar maps. Our research indicates that all four measures are useful in measuring the concept of flourishing. However, they differ from each other depending on specific interest (from a broader to a narrower view). If the aim is to get a comprehensive overview of flourishing, then the PERMA-Profiler, the Mental Health Continuum-Short Form, and the Flourishing Scale are appropriate options. If the focus is on measuring a more central concept, the Wellbeing Conceptual Framework provides the most specific assessment of flourishing.

## 1 Introduction

The issue of assessing wellbeing and mental health of a population has been an important policy indicator for several decades (Beddington et al., 2008; Hapke et al., 2019; Ruggeri et al., 2020). However, even today there are ongoing social and economic inequalities, conflicts, and public health crises, which pose a threat to improving the wellbeing of entire populations (World Health Organization, 2022). As a result, observing and measuring the wellbeing of a population remains a critical socio-political issue that requires further action (Diener and Seligman, 2004; Huppert et al., 2009) to reduce inequalities and ensure equitable flourishing opportunities for all (World Health Organization, 2022). Despite the necessity of wellbeing measurement for public policy, interest in good mental health and wellbeing has not always been the focus of researchers and practitioners. A review of the history of mental health and wellbeing research shows that it took more than half a century for science to move from a psychopathological (mental illness-focused) view to a positive (mental health-focused) approach and to overcome the strict division between these two poles (Ryff and Singer, 1996; Seligman, 2011). It was largely due to the growing academic interest in positive psychology that wellbeing has received more attention (Csikszentmihalyi and Seligman, 2000). Rather than focusing on what is wrong with an individual, positive psychology researchers explore the factors and processes that lead to flourishing (Gable and Haidt, 2005).

Growing interest and research in the field of positive psychology necessitate strong definition and measurement of wellbeing. Despite a rich history of both seminal studies and theories (Argyle and Crossland, 1987; Diener, 1984; Jahoda, 1958; Ryff, 1989; Wilson, 1967) and extensive research (e.g., 164 studies and 560 observations published prior to September 2013; Eger and Maridal, 2015), a lack of consensus remains within the academic community regarding the definition of wellbeing (Dodge et al., 2012; Galderisi et al., 2015; Hone et al., 2014b; Linton et al., 2016; VanderWeele et al., 2020; Willen et al., 2022). Oades and Mossman (2017) aptly summarize that the question of how to define wellbeing easily leads to heated debates and ongoing discussions. While some scholars and organizations, like the World Health Organization (World Health Organization, 2008), claim that mental health and wellbeing are synonymous (e.g., Huppert et al., 2009; Keyes, 2002) others dispute this view (Wren-Lewis and Alexandrova, 2021). According to Wren-Lewis and Alexandrova (2021), mental health is seen as a psychological necessity for the pursuit of any idea of ‘the good life’, including wellbeing, without being the same as wellbeing. However, many researchers agree that wellbeing is considered a dynamic and multifaceted construct (Forgeard et al., 2011; Huppert and Cooper, 2014; Mitchell et al., 2010) in relation to optimal experience and functioning (Ryan and Deci, 2001). Wellbeing surveys that are used for economic and social policies, are often criticized for relying on measures of life satisfaction or happiness that lack reliability due to contextual factors (Huppert et al., 2009). In contrast, wellbeing may best be characterized as a multidimensional construct that cannot be simply measured by a single item (e.g., evaluations of life as a whole; Hone et al., 2014b; Huppert and Cooper, 2014; Huppert and So, 2013).

## 2 Flourishing

The surge in interest in promoting wellbeing has led to many definitions of this concept and a plethora of questionnaires that claim to capture it (Cooke et al., 2016; Lindert et al., 2015; Linton et al., 2016). From a philosophical perspective, two important perspectives on human needs and desires in life have significantly influenced current wellbeing research and measurement: the *hedonic* and the *eudaimonic* tradition (Ryan and Deci, 2001). While both perspectives focus on wellbeing and have some overlap in their understanding of the concept, each perspective emphasizes different aspects. The hedonic tradition emphasizes *subjective wellbeing* (SBW), highlighting the joy and satisfaction of attaining one’s goals to maximize happiness and minimize sorrow (Diener et al., 2009; McDowell, 2010; Mitchell et al., 2010). In contrast, the eudaimonic tradition values *psychological wellbeing* (PWB), that is, virtue, proper behavior, and personal progress toward full functioning (Ryff and Keyes, 1995) which is also associated with flourishing (Fowers et al., 2023). Given these two traditions of research, some researchers include relatively “pure” measures that discriminate or separate across domains when operationalizing wellbeing in their studies (i.e., strictly hedonic: e.g., The Satisfaction with Life Scale; SWLS; Diener et al., 1985; or eudaimonic: e.g., Psychological Wellbeing Scale; Ryff, 1989). Other researchers create composite scores that blend multiple wellbeing domains into a single wellbeing component, such as the Flourishing Scale (FS; Diener et al., 2009).

The concept of flourishing has recently gained prominence partially due to Seligman’s prominent publication stating that flourishing is the ‘gold-standard for measuring wellbeing’ (2011). Others have extended the scope of flourishing beyond mere wellbeing by referring to flourishing as the ‘ultimate end state in psychology’ (Schotanus-Dijkstra et al., 2016) or as ‘complete wellbeing’ (VanderWeele, 2017). Despite relative consensus on its definition, there is debate about what aspects of wellbeing flourishing entails. Most scholars agree that individuals who flourish experience higher levels of both hedonic and eudaimonic wellbeing (Disabato et al., 2016; Schotanus-Dijkstra et al., 2016). As Keyes has argued, hedonic and positive functioning must be present to be classified as a flourishing person (2002). This state can be achieved even by someone without good mental health (Keyes, 2005). Though many seem to agree with this perspective, alternative perspectives argue that flourishing is only related to eudaimonia (see Huta and Waterman, 2014). Taking all of these perspectives into account, the existing literature defines flourishing as the optimal functioning of individuals, organizations, and institutions, characterized by a high level of wellbeing in a person (Butler and Kern, 2016; Hone et al., 2014b; Huppert and So, 2013; Keyes, 2002).

There are currently four widely accepted instruments for measuring flourishing following current scientific literature (Hone et al., 2014b; Pakse and Svence, 2020). These are the Mental Health Continuum-Short Form (MHC-SF; Keyes, 2002), the Flourishing Scale (FS; Diener et al., 2009), the Wellbeing Conceptual Framework (here called WBCF; Huppert and So, 2013), and the PERMA-Profiler (here: PERMA; Butler and Kern, 2016). Even though these questionnaires attempt to measure the same construct, they use different definitions as well as dimensions of flourishing (Hone et al., 2014b). Diener’s FS measures wellbeing as unidimensional, whereas the other three questionnaires conceptualize wellbeing as multi-dimensional. Additionally, Rule et al. (2024) note, that the MHC-SF is the only flourishing scale that has been the subject of a systematic literature review, indicating its extensive use in international wellbeing research. To date, only two studies have compared these four measures of flourishing (Hone et al., 2014b; Pakse and Svence, 2020).

For a short overview of the four measures, see Table 1.

**Table 1:** Instruments to measure flourishing.

Instrument	Subscales	Number of items	Items of each subscale	Hedonic/Eudaimonic
Mental Health Continuum-Short Form (MHC-SF; Keyes, 2002)	Emotional wellbeing (EWB) Social wellbeing (SWB) Psychological wellbeing (PWB)	14	3 EWB 5 SWB 6 PWB	Hedonic and Eudaimonic
PERMA-Profiler (here referred to PERMA; Butler and Kern, 2016)	Positive Emotions (P) Engagement (E) Relationships (R) Meaning (M) Accomplishment (A)	15	3 P 3 E 3 R 3 M 3 A	Hedonic and Eudaimonic
The Flourishing Scale (FS; Diener et al., 2010)	Unidimensional	8	–	Eudaimonic
Wellbeing Conceptual Framework (here referred to WBCF; Huppert and So, 2013)	Positive Appraisal (PA) Positive Characteristics (PC) Positive Functioning (PF)	10	1 PA 5 PC 4 PF	Hedonic and Eudaimonic

### 2.1 Keyes’ definition of flourishing

Based on the Diagnostic and Statistical Manual III-Revised (DSM-III-R; American Psychiatric Association, 1987) and the WHO’s definition of mental health (2004), Keyes’ Mental Health Continuum-Short Form (MHC-SF; Keyes, 2002) defines flourishing as the presence of hedonic and positive functions. The MHC-SF comprises three subscales: psychological, emotional, and social wellbeing. Psychological and social wellbeing pertain to the eudaimonic and emotional wellbeing to the hedonic concept (see Table 1). Emotional wellbeing consists of feelings of happiness, interest in life, and satisfaction, psychological wellbeing of positive functioning in terms of self-actualization, being satisfied with one’s life, and managing responsibilities; and social wellbeing includes social contribution, actualization, and coherence. As suggested by results from the MIDUS survey of 3,032 American adults, mental health is best modeled on two continua (Keyes, 2005). In this view, it is possible to have good wellbeing and function well without having good mental health (Keyes, 2005). In this concept, flourishing refers to people functioning well with high hedonic characteristics, whereas the condition of people with poor mental health is referred to as languishing.

### 2.2 Seligman, and Butler and Kern’s definition of flourishing

According to Seligman (2011), flourishing is seen as the benchmark for measuring wellbeing, and the goal of positive psychology is to increase it. While Seligman (2002) initially postulated three core elements of happiness, namely *positive emotions*, *engagement*, and *meaning*, his later work suggests that these three pillars alone are not sufficient for human flourishing (2011). In his revised model, Seligman included two additional dimensions, *positive relationships,* and *accomplishment,* as pillars (2011). Together, these five pillars form the foundation of flourishing. Based on these five PERMA factors (positive emotions, engagement, positive relationships, meaning, and accomplishment; Seligman, 2011), a brief questionnaire to measure flourishing was developed (Butler and Kern, 2016). In contrast to other models that prioritize either hedonic or eudaimonic wellbeing, the PERMA model incorporates both perspectives. This broad view of wellbeing has influenced both academic and applied psychology, particularly in educational and organizational settings (Wammerl et al., 2019).

### 2.3 Diener et al’s definition of flourishing

The Flourishing Scale (FS; Diener et al., 2010) was created to serve as an addition to other instruments that measure subjective wellbeing (Diener et al., 2009). In developing the FS, Diener et al. (2009, 2010) aimed to create a brief measure that would capture important aspects of human flourishing alongside existing psychological theories, i.e., humanistic theories, social and psychological capital, purpose and meaning, and optimism. Therefore, the FS consists of eight questions that define fundamental characteristics of human functioning, such as positive relationships, feelings of competence, and having meaning and purpose in life (Diener et al., 2010; Esch et al., 2013). Although the authors note that a high score indicates that respondents have a positive self-perception across a range of functional domains, no thresholds are provided for classifying an individual as flourishing.

### 2.4 Huppert and So’s definition of flourishing

After testing the definition of flourishing in the European Social Survey (European Social Survey (ESS), 2006; Round 3 in 2006/7), Huppert and So (2013) used data from 43,000 Europeans to develop The Wellbeing Conceptual Framework (WBCF). Their definition of flourishing considers mental health as the opposite of mental illness. As such, they identified 10 characteristics as opposites to the typical symptoms of anxiety and depression as defined by the DSM-IV and ICD-10: Competence, emotional stability, engagement, meaning, optimism, positive emotions, positive relationships, resilience, self-esteem, and vitality (Huppert and Cooper, 2014; Huppert and So, 2013). According to Huppert and So’s model, participants must score on an indicator of positive emotion and three or four components of positive functioning or positive characteristics to be classified as flourishing (Hone et al., 2014b). As with the MHC-SF and the PERMA-Profiler, this questionnaire assesses both aspects of wellbeing, namely eudaimonia and hedonia. Further research led to the development of the Wellbeing Profile (WB-Pro; Marsh et al., 2020), which expanded the characteristics of the WBCF from 10 to 15. However, to maintain consistency with past research (Hone et al., 2014b; Pakse and Svence, 2020), this study follows the original WBCF framework.

Although these four operational definitions of flourishing might differ in their theoretical underpinnings, they have two things in common, namely that flourishing is associated with high levels of subjective wellbeing, and that wellbeing is a multidimensional construct that cannot be adequately measured by only a single item assessment (Hone et al., 2014b).

## 3 The present study

With the increasing incidence of mental health problems such as depression and anxiety (Brooks et al., 2020; Herrman et al., 2022), an abundance of theories and questionnaires are available to assess wellbeing. However, the arbitrary use of different wellbeing measures poses a challenge, as different studies assess the concept of wellbeing differently (Ackerman et al., 2018; Diener and Seligman, 2004; Petras et al., 2024).

In this context, the ‘jingle jangle’ problem (e.g., Flake and Fried, 2020; Marsh et al., 2020) is a key issue for researchers and practitioners when choosing an appropriate measure. The *jingle* (Thorndike, 1904) fallacy occurs when two measures have the same name but assess different constructs. For example, two measures may both include the term ‘wellbeing’ in their names, but one may assess flourishing while the other measures subjective wellbeing. In contrast, the *jangle* (Kelley, 1927) fallacy occurs when two measures with different names actually assess the same construct, such as when one measure uses the term ‘happiness’ while another uses the term ‘wellbeing’, but both measure the concept of flourishing. Thus, due to the plethora of studies (e.g., 99 questionnaires claiming to assess wellbeing; Linton et al., 2016) and varying definitions choosing an appropriate measure can be therefore quite challenging. Therefore, researchers and clinicians need to carefully consider the specific constructs that each questionnaire measures before choosing the most appropriate one for their purposes.

A similar issue arises with questionnaires that intend to assess flourishing. An initial study comparing the prevalence of flourishing in New Zealand (Hone et al., 2014b) revealed significant differences in prevalence rates depending on the measure of flourishing used. Nevertheless, strong agreement was found between the MHC-SF (Keyes, 2002) and the PERMA-Profiler (Butler and Kern, 2016), as well as moderate agreement between the PERMA-Profiler and the WBCF (Huppert and So, 2013). A later study with a Latvian sample (Pakse and Svence, 2020) examined psychometric validity of the four measures, finding that the flourishing scores of the four measures were all positively correlated with each other (*r* = 0.50–0.70). Such high levels of convergent validity suggest that all four measures may be interchangeable. Despite this heartening evidence, it remains unclear whether they truly assess the same concept of flourishing. Accordingly, the question of which measure is optimal for assessing flourishing remains unresolved.

To address this issue, a new approach called Item Pool Visualization (IPV; Dantlgraber et al., 2019), which is based on Structural Equation Modeling (SEM), is adopted for the purpose to compare methodological foundations of all four measures. SEMs are typically used in the health sciences to determine how components cluster and how much additional information they provide within a factor (Huppert and Cooper, 2014). By providing a convenient way to compare the similarities and differences of multiple questionnaires on a common construct, the IPV (Dantlgraber et al., 2019) offers an advancement over a single SEM model. Unlike SEM, IPV uses two SEMs in combination. First, a diagram approach to combine items and item pools (scales) from different psychological tests using a single data set (Dantlgraber et al., 2019; Petras et al., 2024). Second, a single-factor SEM is calculated in which all items from all measures load on a single factor. This model represents the ‘core’ (i.e., center) of the construct in question (in the present case, flourishing). Therefore, each measure method is represented by ray-structured item pool visualizations. Based on the distance to the center of the first SEM, IPV displays which item sub-pools (or individual items) are closer to the core of the concept (i.e., flourishing) and which are further from the center and therefore less representative of the underlying concept.

Previous research employing the IPV method has provided valuable insights into a range of constructs, including of *self-esteem* (Dantlgraber et al., 2019), *(positive) body image* (Swami et al., 2020), and *interoceptive sensibility* (Todd et al., 2022) by illuminating the challenges of handling complex data and measuring it with a variety of instruments. Building on this work, the present study seeks to extend existing research on wellbeing by conducting a comparative analysis of the items and scales of four widely used self-report instruments that claim to measure the construct of flourishing. The objective is therefore not only to contribute further to IPV research, but also to investigate whether all four instruments are assessing the same construct.

## 4 Method

### 4.1 Participants

All participants (*N* = 698) were German-speaking adults (74.2% from Austria, 24.8% from Germany, 0.3% from Switzerland, and 0.4% from other countries, namely one from Hungary, one from the Netherlands and one who did not specify the country, and 0.3% missing). Participants were mainly women (71.6%; 26.8% men, 1.3% other, and 0.3% missing) and aged 18 to 79 years (*M* = 33.86, *SD* = 13.42; 4 missing). According to Dyussenbayev’s (2017) classification, the age groups are as follows: 35.88% (18–24 years), 40.06% (25–44 years), 20.17% (45–60 years), 3.75% (61–75 years) and 0.14% (76–90 years). Most participants were in a relationship (38.5%; 26.4% married, 32.4% single, 2.4% divorced, and 0.3% widowed). 39.4% had a high school diploma, 42.6% had a university degree, 13.5% had an apprenticeship degree, and 4.5% had attended compulsory school.

### 4.2 Measures

Four measures widely used to assess flourishing were employed based on current scientific literature, namely the Mental Health Continuum-Short Form (MHC-SF; Keyes, 2002, 2009), the Flourishing Scale (FS; Diener et al., 2009), the Wellbeing Conceptual Framework (here referred to WBCF; Huppert and So, 2013), and the PERMA-Profiler (here referred to PERMA; Butler and Kern, 2016). Further, participants were asked to provide information on their gender (female, male, other), age, current relationship status, and highest level of education as demographic items.

#### 4.2.1 The mental health continuum-short form

Derived from the 40-item Mental Health Continuum-Long Form (MHC-LF; Keyes, 2002; Keyes, 2005), the 14-item Mental Health Continuum-Short Form (MHC-SF; Keyes, 2002; Keyes et al., 2008; German form: Żemojtel-Piotrowska et al., 2018) assesses a person’s mental wellbeing over the previous month. The MHC-SF assumes three dimensions of flourishing, *emotional* (EWB), *psychological* (PWB), and *social wellbeing* (SWB). Items are rated on a 6-point scale, ranging from 0 (*never*) to 5 (*every day*). The MHC-SF is considered as a reliable and valid measure of mental health (Iasiello et al., 2022; Lamers et al., 2011) and has gained popularity over the years, having been used worldwide in different languages and different cultures (e.g., French: Doré et al., 2017; Chinese: Guo et al., 2015; German: Stieger et al., 2023; Żemojtel-Piotrowska et al., 2018).

#### 4.2.2 The PERMA-Profiler

The PERMA-Profiler (PERMA; Butler and Kern, 2016; German form: Wammerl et al., 2019) consists of 23 items divided into five facets. Three items measure each of the five criteria of PERMA (*Positive Emotions, Commitment, Relationships, Meaning,* and *Achievements*), and an additional eight items assess *general health, negative emotions, loneliness,* and *happiness* (Butler and Kern, 2016; Wammerl et al., 2019). A large-scale validation study on English-speaking subjects showed high reliability, temporal stability, and construct validity (Butler and Kern, 2016). Additionally, an acceptable to good internal consistency could be shown for all overall PERMA-Profiler scores and its subscales (Butler and Kern, 2016; Ryan et al., 2019; Seligman, 2018; Wammerl et al., 2019). Although it has response categories with a consistent 11-point Likert scale, the scale anchors vary (*never* to *always*; *terrible* to *excellent*; *not at all* to *completely*). Butler and Kern’s (2016) calculation of overall wellbeing was based on the mean of the 15 PERMA items and a single happiness item. However, Bartholomaeus et al. (2020) argue against the use of a single happiness item, as this would not be compatible with the PERMA theory and the factor structures tested in their study. Therefore, in line with Bartholomaeus et al. (2020), this study used the 15 items version in line with the PERMA theory instead of all 23 items.

#### 4.2.3 The flourishing scale

The 8-item Flourishing Scale (FS; Diener et al., 2010; German form: Esch et al., 2013), derived from the 12-item Psychological Flourishing Scale, assesses flourishing while providing a single score for this construct. Items are rated on a 7-point scale, ranging from 1 (*strongly disagree*) to 7 (*strongly agree*), and summed up with higher scores reflecting psychological resources and strengths. The validity, reliability, and one-factor structure of the 8-item FS have been confirmed in different populations in different languages and countries (e.g., Germany: Esch et al., 2013; Italy: Giuntoli et al., 2017; New Zealand: Hone et al., 2014a; Portugal: Silva and Caetano, 2013).

#### 4.2.4 The wellbeing conceptual framework

The wellbeing Conceptual Framework (WBCF; Huppert and So, 2013; German form: ESS Round 3, wellbeing Module; 2006) consists of 10 items with different response categories, with most items rated on a 5-point Likert scale (0 = *strongly disagree*; 4 = *strongly agree*), emotional stability and vitality rated on a 4-point Likert scale (0 = none *or almost none of the time*; 3 = *all or almost all of the time*), and positive emotions rated on an 11-point Likert scale (0 = *extremely unhappy*; 10 = *extremely happy*). In addition, Pakse and Svence (2020) reported good internal consistency for the WBCF and high convergent validity with other flourishing measures used in their study.

In their seminal publication Huppert and So (2013) proposed both a two-factor and a three-factor model for the WBCF. While both models showed a good fit, the minimal change in the fit indices for the two-factor model was highlighted as a positive indicator, making it a slightly more robust choice (Huppert and So, 2013). In line with previous research (for similar reasoning, see Pakse and Svence, 2020), this study focuses on the two-factor solution which divides flourishing into *positive characteristics* (emotional stability, vitality, optimism, resilience, positive emotions, and self-esteem) and *positive functioning* (engagement, competence, meaning, and positive relationships).

### 4.3 Procedure

The questionnaire took 10–15 min to complete and was created via the software *LimeSurvey* which was locally installed at the first and last authors’ university. The only requirement for participation was the minimum age of 18 years. Data were collected in two waves: the first wave from December 2022 to February 2023, and the second wave from April to June 2023. The study’s sample was obtained through a snowball approach using various social media platforms including Facebook, Twitter, and Instagram. Furthermore, emails were sent to psychology students enrolled at the university. After completing a digital consent form, participants were asked to complete an anonymous questionnaire containing the measurement tools and demographic information. To increase participant engagement and commitment, a pop culture quiz was integrated into the data collection procedure, as in previous studies (e.g., Stieger et al., 2023). In the current project, we based the survey on the popular television series *The Simpsons*. In this way, participants received personalized feedback on their wellbeing and to which Simpsons character this wellbeing score is most similar. A preliminary study (*N* = 56) examined the wellbeing of the series’ central characters (Homer, Marge, Lisa, Bart, Maggie, and Flanders) using the five PERMA attributes and the FS. This preliminary survey provided valuable insights into the characters’ overall wellbeing (e.g., Maggie was perceived as the character with the highest wellbeing score).

For participants to obtain valid and reliable personal feedback at the end of the questionnaire, it was chosen to implement a forced-response approach to avoid missing data. A total of 1,603 people visited the info page of the study, of which 1,172 (73.1%) agreed to participate. Of those who agreed, 698 (59.5%) completed the entire online questionnaire with no missing data across the four flourishing scales. Thirty participants dropped out after completing the MHC-SF, while 474 dropped out earlier, mostly before consent, as the forced response approach was not applied to those who left the survey early.

### 4.4 Statistical analyses

#### 4.4.1 Preliminary analyses

To examine the fit of the higher dimensional models for the four measures in our dataset, confirmatory factor analyses (CFA) were computed using the *lavaan* package (Rosseel, 2012) in R (R Core Team, 2021). This is not a required step in the IPV but may be useful in helping to make sense of IPV results (see also, Swami et al., 2020). Testing the data for normality revealed that they were mostly not univariate normal distributed, so parameter estimates were obtained using the robust maximum likelihood method with the Satorra-Bentler correction (Satorra and Bentler, 2001) in line with past IPV studies (e.g., Todd et al., 2022).

The chi-square of the normalized model (χ^2^/df), the Steiger-Lind root mean square error of approximation (RMSEA) and its 90% confidence interval (CI), the standardized root mean square residual (SRMR), and the comparative fit index (CFI) were used to assess goodness of fit. Regarding the normalized model chi-square, values less than 3.0 are seen as an indicator of a good fit, and values up to 5.0 are considered reasonable (Hu and Bentler, 1999). For the RMSEA, values close to 0.06 are considered an indicator of a good fit, and values of about 0.07–0.08 are considered an indicator of a moderate fit (Steiger, 2007). For the SRMR, values less than 0.09 are indicators of an appropriate fit (Hu and Bentler, 1999) while the CFI values close to or greater than 0.95 are indicators of an appropriate fit (Hu and Bentler, 1999).

#### 4.4.2 Item pool visualization

To perform the IPV analyses, the *IPV* package (Petras and Dantlgraber, 2021) in R (R Core Team, 2021) was used. As a fundamental basis for the IPV analyses, SEMs were calculated using the *lavaan* package (Rosseel, 2012). In line with previous studies (Dantlgraber et al., 2019; Swami et al., 2020; Todd et al., 2022), the following steps were carried out. First, a general factor model was estimated based on Dantlgraber et al. (2019). For this purpose, a single factor was extracted from the item pool, which in this case contains all flourishing items that represent the core of the flourishing concept. Then, in a second step, factors were extracted from smaller but more precise sub-pools to create a correlated factor model. For example, for the MHC-SF, three sub-pools were extracted according to Keyes (2002, 2018) definition: social, emotional, and psychological wellbeing. In addition, center distances were calculated, distinguishing between normal center distances and aggregate center distances of item sub-pools. Center distances indicate how much better individual items are explained by specific factors compared to the general factor. Aggregate center distances, also known as mean center distances, summarize this increase in explained variance across all items in a sub-pool. This IPV specific method stands for the proportional increase in explained item variance, whereas a center distance of zero represents no increase. Therefore, a center distance close to zero indicates that an item (or combination of items) is strongly related to the central core concept, whereas a larger center distance indicates that an item, or groups of items measure a component of the construct that is more distant (or even not included) in the main concept.

## 5 Results

### 5.1 Preliminary analyses

The results of the CFAs are summarized in Table 2.

**Table 2:** Results of confirmatory factor analyses of all four flourishing measures examining the fit of the models.

Scale	SBχ^2^	df	χ^2^/df	Robust RMSEA (90% CI)	SRMR	Robust CFI
(1) MHC-SF	265.09	74	3.58	0.069 (0.060, 0.078)	0.062	0.950
(2) PERMA	280.74	80	3.51	0.072 (0.063, 0.081)	0.037	0.957
(3) FS	93.50	20	4.68	0.094 (0.075, 0.114)	0.038	0.951
(4) WBCF	118.85	34	3.50	0.065 (0.053, 0.078)	0.043	0.950

CI, confidence interval; MHC-SF, mental health continuum–short form; FS, flourishing scale; PERMA, perma-profiler; WBCF, the wellbeing conceptual framework. SBχ^2^, Satorra-Bentler chi-square (adjusted for non-normality); df, degrees of freedom; χ^2^/df, Chi-square divided by degrees of freedom; RMSEA, root mean square error of approximation (fit index); SRMR, standardized root mean square residual; CFI, comparative fit index. All *p*s < 0.001.

The fits of the factor models for all measures indicated a moderate fit (all χ^2^/df ranging from 3.50 to 4.68). Regarding RMSEA, except for FS (0.09), MHC-SF, PERMA, and WBCF showed an acceptable fit. The SRMR was less than 0.09 in all four questionnaires, i.e., acceptable. Robust CFI, then, was also acceptable (above 0.95) for all measures. Overall, the results of all instruments had adequate internal consistency coefficients as measured by Cronbach *α* (all α > 0.61; see Table 3).

**Table 3:** Internal consistency coefficients, descriptive statistics, and intercorrelations between scores on all flourishing measures.

Scale	# Items	*M* (*SD*)	(1)	(2)	(3)	(4)	(5)	(6)	(7)	(8)	(9)	(10)	(11)
(1) MHC-EWB	3	4.56 (1.06)	*0.84*										
(2) MHC-SWB	5	3.37 (1.31)	0.57	*0.81*									
(3) MHC-PWB	6	4.42 (1.04)	0.78	0.64	*0.86*								
(4) PERMA-P	3	6.57 (1.93)	0.80	0.55	0.75	*0.91*							
(5) PERMA-E	3	6.87 (1.67)	0.46	0.28	0.45	0.52	*0.61*						
(6) PERMA-R	3	7.39 (2.01)	0.61	0.48	0.64	0.72	0.38	*0.81*					
(7) PERMA-M	3	6.92 (2.08)	0.68	0.56	0.75	0.78	0.48	0.67	*0.89*				
(8) PERMA-A	3	6.71 (1.71)	0.63	0.47	0.71	0.74	0.48	0.57	0.77	*0.80*			
(9) FS	8	5.45 (0.94)	0.65	0.57	0.73	0.72	0.43	0.63	0.75	0.71	*0.89*		
(10) WBCF-PC	6	> −0.01 (0.69)	0.73	0.51	0.72	0.79	0.43	0.55	0.67	0.69	0.68	*0.78*	
(11) WBCF-PF	4	3.15 (0.59)	0.63	0.55	0.71	0.68	0.48	0.58	0.75	0.66	0.71	0.59	*0.68*

# Items, number of items; MHC-SF, Mental Health Continuum–Short Form; MHC-SF EWB, Emotional Well-Being; MHC-SF SWB, Social Well-Being; MHC-SF PWB, Psychological Well-Being; PERMA, Perma-Profiler; PERMA-P, Positive Emotions; PERMA-E, Engagement; PERMA-R, Positive Relationships; PERMA-M, Meaning; PERMA-A, Accomplishment; FS, Flourishing Scale; WBCF, The Well-Being Conceptual Framework; WBCF-PC, Positive Characteristics; WBCF-PF, Positive Functioning. WBCF-PC is standardized because of different Likert scales. All correlations were significant at *p* < 0.05. Diagonal entries = Cronbach α.

One exception is the PERMA-E with low reliability (0.61), but this is also in line with past research (e.g., 0.58; Bartholomaeus et al., 2020).

### 5.2 Item pool visualization

#### 5.2.1 Item-based analyses

**Figure 1:**
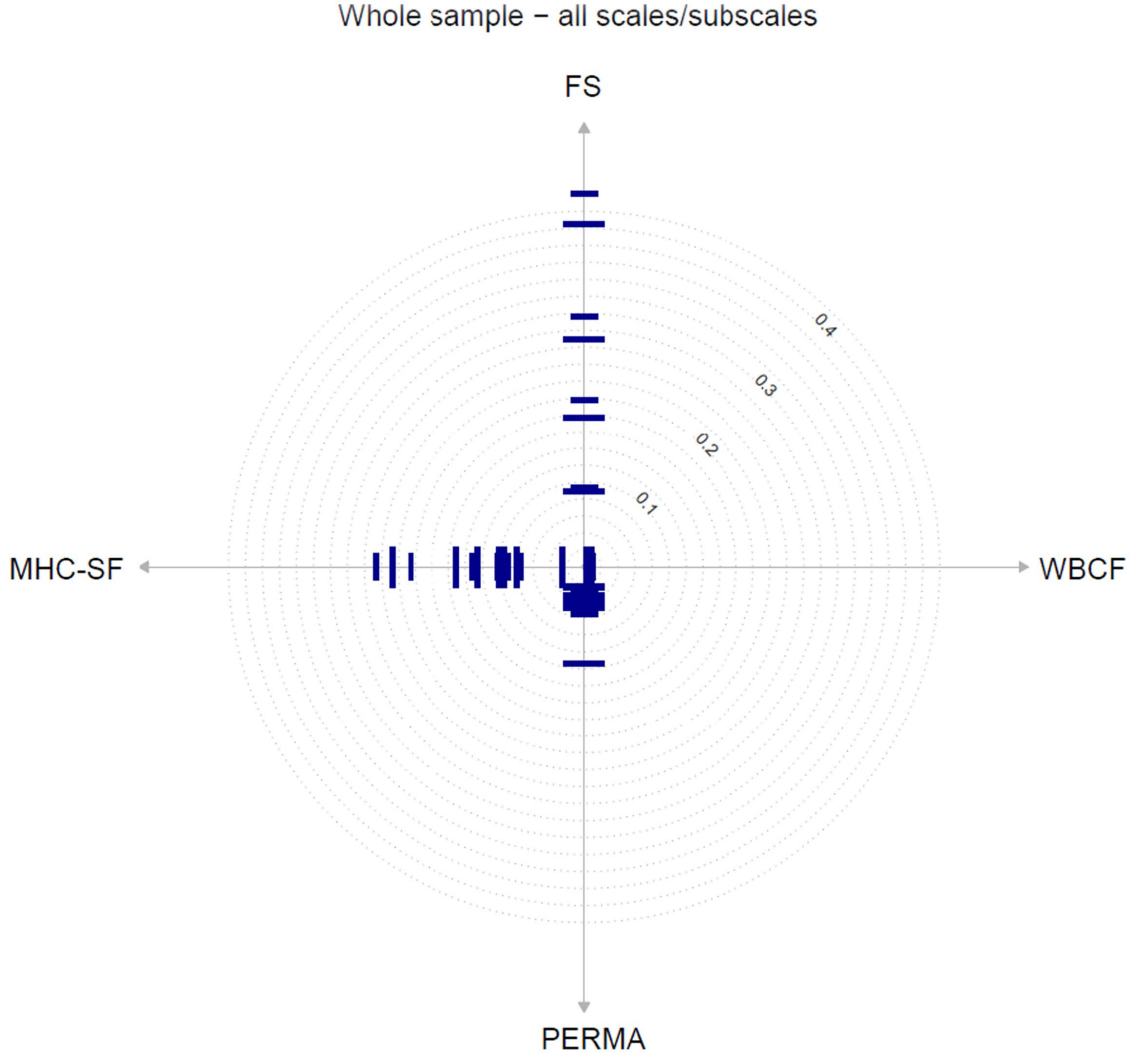
Radar charts with item locations on scale dimensions for all measures. The dotted circles represent the grid of axis scaling. For clearer distinction, every second item is illustrated as having a different length. MHC-SF, Mental Health Continuum - Short Form; PERMA, Perma-Profiler; FS, Flourishing Scale; WBCF, The Well-Being Conceptual Framework.

For each of the four flourishing measures (i.e., item pools), IPV calculates the mean center distance of all items on the respective scale, with a small deviation indicating that all items are constructed similarly. Compared to the other measures (WBCF and PERMA), the item-based analyses showed that the FS and the MHC-SF had the most heterogeneous item sets (see Figure 1).

In terms of the MHC-SF, subscale SWB4 (“During the past month, how often did you feel confident to think or express your own ideas and opinions?,” center distance = 0.24) had the largest distance from the center, followed by item SWB5 (“During the past month, how often did you feel that the way our society works made sense to you,” center distance = 0.22) and item SWB3 (“During the past month, how often did you feel that our society is a good place, or is becoming a better place, for all people,” center distance = 0.20). Across all PERMA items, item PERMA-E3 (“How often do you lose track of time while doing something you enjoy?,” center distance = 0.11) showed the greatest distance from the core construct. The items that were most central within the entire item pool were all WBCF items and most of the PERMA items (center distances between 0.02 and 0.05). Four of the FS items were far from the center (>0.20), two of which were above 0.40, which were item FS4 (“That you had something important to contribute to society,” center distance = 0.44; see Table 4) and item FS5 (“That you belonged to a community,” center distance = 0.40) were therefore deviating the furthest from the center (=core construct flourishing).

**Table 4:** Basic item pool visualization calculations for the nested model.

Scale	Item #	Factor loadings		Center distance	Mean center distance/Aggregate center distance
		General factor model	Correlated factor model		
MHC-SF-EWB	1	0.677	0.700	0.072	0.122/0.104
MHC-SF-EWB	2	0.717	0.760	0.123	
MHC-SF-EWB	3	0.790	0.824	0.087	
MHC-SF-SWB	1	0.575	0.600	0.091	
MHC-SF-SWB	2	0.520	0.558	0.148	
MHC-SF-SWB	3	0.474	0.520	0.203	
MHC-SF-SWB	4	0.450	0.502	0.243	
MHC-SF-SWB	5	0.460	0.509	0.223	
MHC-SF-PWB	1	0.710	0.744	0.099	
MHC-SF-PWB	2	0.692	0.718	0.077	
MHC-SF-PWB	3	0.658	0.689	0.097	
MHC-SF-PWB	4	0.567	0.602	0.128	
MHC-SF-PWB	5	0.584	0.613	0.098	
MHC-SF-PWB	6	0.811	0.820	0.023	
PERMA-A	1	0.800	0.811	0.028	0.039/0.033
PERMA-A	2	0.676	0.688	0.038	
PERMA-A	3	0.592	0.601	0.031	
PERMA-E	1	0.429	0.440	0.054	
PERMA-E	2	0.579	0.587	0.030	
PERMA-E	3	0.260	0.274	0.111	
PERMA-P	1	0.818	0.833	0.038	
PERMA-P	2	0.819	0.833	0.034	
PERMA-P	3	0.861	0.871	0.021	
PERMA-M	1	0.841	0.850	0.022	
PERMA-M	2	0.791	0.799	0.020	
PERMA-M	3	0.785	0.801	0.041	
PERMA-R	1	0.579	0.591	0.041	
PERMA-R	2	0.655	0.665	0.032	
PERMA-R	3	0.668	0.683	0.045	
FS	1	0.757	0.790	0.090	0.243/0.217
FS	2	0.548	0.599	0.195	
FS	3	0.699	0.757	0.173	
FS	4	0.520	0.623	0.438	
FS	5	0.576	0.682	0.402	
FS	6	0.694	0.780	0.266	
FS	7	0.727	0.758	0.086	
FS	8	0.561	0.638	0.294	
WBCF-PC	1	0.691	0.688	0.007	0.001/0.000
WBCF-PC	2(R)	0.173	0.170	0.001	
WBCF-PC	3	0.679	0.678	0.000	
WBCF-PC	4	0.473	0.475	0.000	
WBCF-PC	5	0.570	0.570	0.007	
WBCF-PC	6	0.810	0.813	0.000	
WBCF-PF	1	0.690	0.688	0.000	
WBCF-PF	2	0.351	0.351	0.000	
WBCF-PF	3	0.646	0.643	0.000	
WBCF-PF	4	0.572	0.571	0.000	

Item numbers followed by (R) were reverse scored before analysis. MHC-SF, mental health continuum–short form; MHC-SF-EWB, emotional wellbeing; MHC-SF-SWB, social wellbeing; MHC-SF-PWB, psychological wellbeing; PERMA, perma-profiler; PERMA-P, positive emotions; PERMA-E, engagement; PERMA-R, positive relationships; PERMA-M, meaning; PERMA-A, accomplishment; FS, flourishing scale; WBCF, the wellbeing conceptual framework; WBCF-PC, positive characteristics; WBCF-PF, positive functioning.

In summary, WBCF and PERMA tend to assess the core concept of flourishing, while MHC-SF and FS show more distant (or broader) aspects of the central construct. Nevertheless, all center distances were relatively low (<0.44), compared to recent literature (Swami et al., 2020: center distance <8.60; Todd et al., 2022: center distance <13.65), meaning, although there were differences across the flourishing measures, based on the absolute value of the center distances, all of them assessed the core of flourishing very well.

#### 5.2.2 Scale-based analyses

**Figure 2:**
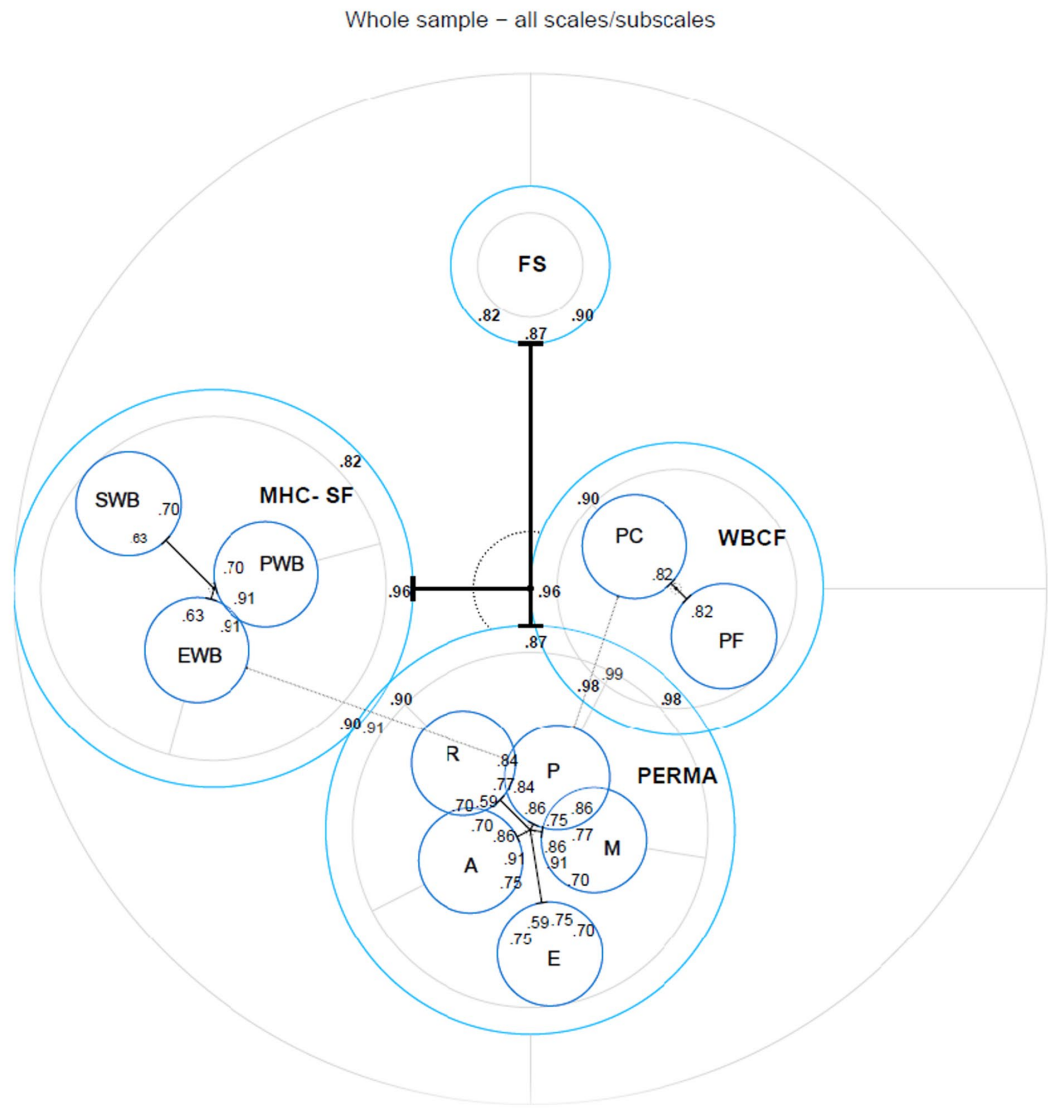
A nested model to demonstrate center distances within all scales and across all the measures of flourishing. The position of the subscales within the subscales cannot be interpreted relative to the other measures in the chart. MHC-SF, mental health continuum–short form; EWB, emotional wellbeing; SWB, social wellbeing; PWB, psychological well-being; PERMA-P, positive emotions; PERMA-E, engagement; PERMA-R, positive relationships; PERMA-M, meaning; PERMA-A, accomplishment; FS, flourishing scale; WBCF, the wellbeing conceptual framework; WBCF-PC, positive characteristics; WBCF-PF, positive functioning.

For each of the measures IPV center distances were generated. Examining the scale view (see Figure 2), each item pool is displayed as a circle, while the values within the circles are the correlations (standardized path coefficients) between the measures.

The WBCF can be seen to be most strongly associated with the center (showing the lowest center distance), followed by the PERMA and the MHC-SF. Of all the measures, the FS was the furthest from the center. In addition to the scale-specific circles in Figure 2, latent correlation values were found, with all measure methods correlating well with each other, but the WBCF appears to have the highest latent correlations with all other measure methods (0.90–0.99). In conclusion, all the questionnaires were highly correlated with each other, and all were centrally located.

Furthermore, the subscales and, therefore, the center distances within each instrument can be examined. On the MHC-SF, the psychological wellbeing and emotional wellbeing subscales were positioned in the middle with minimal differences in center distance, whereas the social wellbeing subscale was the most distant. When analyzing the PERMA subscales, it was found that the engagement subscale had the biggest distance from the center, while the positive emotions, involvement, positive relationships, meaning, and accomplishment subscales were located closest. The WBCF’s positive characteristics and positive functioning, however, showed mixed distances from the center, ranging from very central to further out.

## 6 Discussion

The current study aimed to contribute to a deeper understanding of the measure of flourishing and to provide guidance on the selection of appropriate instruments for both research and practical application (as recommended in Forgeard et al., 2011). Building on existing research (Swami et al., 2020; Todd et al., 2022), we examined the similarities and distinctiveness of four self-reported flourishing measures (following Hone et al., 2014b; Pakse and Svence, 2020) using Item Pool Visualization (IPV) to determine if these measures load onto a global flourishing factor and whether some of these measures were more central to the core construct than others.

In our study, all four measures had items loaded on a general component, which led to reasonably close center distances. This is congruent with Pakse and Svence’s (2020) research, as all four measures were found to be strongly associated with each other and therefore appear to measure the same construct (i.e., flourishing). The Wellbeing Conceptual Framework (WBCF) provided the most accurate measure of the core construct, while the Flourishing Scale’s (FS) items showed the greatest variance among the four measures. At first glance, this variability of the FS may seem irrelevant to the core assessment of flourishing. However, it can be interpreted in two ways: It could provide a deeper understanding of the concept used in this study or it could indicate potential problems with the validity of the FS, suggesting that it may capture fewer central aspects of the construct. However, according to Swami et al. (2020), such diverse items do not inherently undermine the usefulness of the measure, as there may be specific cases where researchers may wish to use a “broader” instrument such as the FS.

Concerning the MHC-SF, the original version postulates a three-factor structure consisting of three types of wellbeing (emotional, psychological, and social). However, this three-factor structure has been repeatedly discussed and questioned in the literature. Results from a recent study in Australia and Singapore (Yeo and Suárez, 2022) indicate that the best fit to data from two samples was a bi-factorial model of the MHC-SF. The authors also note the unidimensionality of the measure, suggesting that interpreting the aggregate general score of the MHC-SF to be more informative than independent factor scores taken separately. Looking at Figure 2, it appears that the MHC-SF and its 3-dimensional structure may not be the best fit as EWB and PWB are quite central to the core construct of the MHC-SF.

Regarding the PERMA-Profiler, two points warrant discussion. The first concerns the engagement (E) Factor, while the second relates to the construction of the response scales. Looking at the item analysis, it is apparent that the PERMA-E factor was found to be the furthest from the center within the PERMA scale, supporting prior research (Bartholomaeus et al., 2020; Goodman et al., 2018) that found engagement to be the factor that had the weakest correlation with most other PERMA variables. As the reliability of this subscale was also comparatively low, further examination of the psychometric data of this measure might be needed.

As has been pointed out in the literature (Bartholomaeus et al., 2020; Reise et al., 2013), questionnaires are repeatedly fraught with methodological flaws, starting with the construction of the response scales. The PERMA for instance, uses different response scale anchors that vary from item to item. Additionally, there are also problems with the theoretical background of the PERMA. For instance, in Seligman’s PERMA model, happiness is not included as a variable in the experience of flourishing, but it is included in the PERMA along with the other 15 PERMA components in the calculation of the overall wellbeing score. However, strictly following the original theory, it should not be part of the total score (Bartholomaeus et al., 2020), as it has been in other studies (Butler and Kern, 2016). Furthermore, this leads to another issue of the PERMA, as the number of PERMA elements used in the literature is not always the same. For example, Pakse and Svence (2020) included the entire PERMA-Profiler (i.e., 23 items) in their study, while Hone et al. (2014b) used 16 items from the PERMA and excluded general health, negative emotions, and the loneliness item. However, following Bartholomaeus et al. (2020), this study used the original 15 items of the PERMA theory which is in line with Seligman’s PERMA (= flourishing) concept.

The subscales of all measures (except FS) are highly intercorrelated (0.83–0.99), whereas the WBCF correlates very highly with all of them (with FS: 0.90, with MHC-SF: 0.96; with PERMA: 0.99). Given the high level of agreement between the measurement instruments, it can be assumed that despite having different names and approaches, they essentially measure the same underlying construct.

Due to the results of the IPV, the current factor structure of the three questionnaires (FS is excluded as it is unidimensional) seems to be questionable. Beginning with the WBCF, the most recent version of the WBCF (Ruggeri et al., 2020) uses three factors, whereas other studies such as Pakse and Svence (2020) use a two-factor model. Given the high intercorrelation of the items, it is questionable whether three or even two factors are necessary or whether only a unidimensional factor design is sufficient. However, additional analyses with the present data did show a significantly better fit of the suggested two-factor solution compared with a unidimensional factor design (RMSEA = 0.08; Table S1 in the online supplementary; two-factor solution: RMSEA = 0.07; Table 2).

There is also the question of whether a meaningful distinction can be made between hedonic and eudaimonic items or whether they assess the same concept of wellbeing. In their study, Goodman et al. (2018) found a high degree of similarity between the PERMA-Profiler and subjective wellbeing (SWB), with a latent correlation coefficient of 0.98.

### 6.1 Limitations

Although consistent with previous IPV studies (*N* = 501; Swami et al., 2020; *N* = 802, Todd et al., 2022), our sample may not be fully representative of the general population, as it is predominantly German-speaking women with higher levels of education. However, this limitation, is not expected to have a significant impact on the results, particularly regarding the aggregate center distances of the measures. Consequently, although the sample may not be suitable for validation studies, it is considered adequate for correlational analyses as a community-based sample.

Another potential limitation may arise from the questionnaires used. First, the questionnaires in this study were based on previous literature (Hone et al., 2014b; Pakse and Svence, 2020), and excluded newer questionnaires (see Rule et al., 2024; Willen et al., 2022) such as the Flourishing Index (Węziak-Białowolska et al., 2019) the WB-Pro (Marsh et al., 2020), or the wellbeing assessment (WBA; Węziak-Białowolska et al., 2021). Furthermore, IPV defines the core concept to be measured as the sum of all items from measures that load on a single factor. However, one disadvantage to this approach is that if all the measures do not actually assess flourishing, then the core cannot represent flourishing either. Adding another measure to the scale could therefore change the central core. Nevertheless, since our analysis is based on 47 items, it is expected to be stable. Therefore, adding another measure is unlikely to change the core significantly.

An additional issue relates again to the observed center distances in this study, as they are significantly smaller than those reported in previous IPV-based studies (Swami et al., 2020; Todd et al., 2022). For example, Todd et al. (2022) reported a maximum center distance of 13.65 in their study, which is 34 times higher than the maximum center distance (cd = 0.40) in the current study. Although the center distances in this study may appear large, especially when looking at the illustrations of the IPV, they are not when compared to those in other IPV studies. However, this also means that all four measures and their items in this study are closely centered around the construct of flourishing, indicating a reasonable and coherent representation of flourishing, despite the (small) differences in item distances.

### 6.2 Practical implications

Although there is a substantial body of evidence on appropriate measures of wellbeing (see VanderWeele et al., 2020 for a comprehensive review), there remains a lack of methodologically accurate and well-validated measures. Many existing questionnaires contain redundant items, suggesting a need for refinement rather than developing new scales. Our study found that items across four questionnaires effectively measured the core construct of wellbeing, as evidenced by small center distances and high inter-item correlations. This redundancy highlights the need for more efficient measures.

A significant challenge in wellbeing research is the jingle-jangle fallacy (Flake and Fried, 2020; Marsh et al., 2020), where scales purport to measure similar constructs under different names or different constructs under the same name. This issue, observed in various domains such as depression (Fried, 2017) and mindfulness (Altgassen et al., 2024), leads to theoretical confusion and threatens research validity (Hanfstingl et al., 2024). To overcome the jingle-jangle fallacy in the construct of wellbeing in future research, we would like to refer to Hanfstingl et al. (2024) and their call for the collaborative efforts within the scientific community. Through expert group discussions, researchers can refine definitions and avoid construct mislabeling, ultimately improving the consistency and validity of wellbeing measures.

Overall, the results of this study have important practical implications for both researchers and practitioners. Our analysis suggests that well-established flourishing measures such as the WBCF, PERMA, MHC-SF, and FS are useful for assessing different aspects of wellbeing. Given that these measures were highly correlated in our study and captured the core construct of flourishing, we can recommend the use of all of them. Thus, from a practical perspective, the PERMA-Profiler and the MHC-SF provide broad assessments, making them ideal for large-scale public health interventions or policies aimed at improving overall wellbeing. In contrast, the WBCF provides a more focused assessment of the core construct, which is particularly useful in clinical settings where a detailed understanding of flourishing is needed to adapt interventions for individuals or specific groups.

## 7 Conclusion

The aim of this study was to compare and contrast four prominent measures of flourishing using Item Pool Visualization (IPV), as selecting an appropriate flourishing measure can be challenging due to the increasing number of theories and measures available (see a recent review of eight flourishing measures in Fowers et al., 2023). The WBCF, with only 10 items, captures the concept of flourishing very well. Because of the high correlation between the items, it could be reconsidered to remove some of the similar (highly correlated) items to create a brief and useful questionnaire.

Just as mental wellbeing is a lifelong concern, it is essential for countries to implement early intervention strategies that support both individual health and economic prosperity (Beddington et al., 2008; Herrman et al., 2022). Wellbeing questionnaires are crucial not only for policy purposes, but also in the field of clinical psychology, where they allow the assessment of individual wellbeing and help to prevent future risks of mental illness. The question that arises is which of these flourishing questionnaires should be chosen by a researcher or practitioner. To answer this question, Forgeard et al.'s (2011) metaphor of wellbeing measures being compared to a car’s performance is helpful. While some people are only interested in understanding how the car performs overall, others seek to understand how individual components work and why. This analogy can be applied to the current study. For a broad assessment of flourishing, the PERMA-Profiler, the Flourishing Scale (FS), and the Mental Health Continuum-Short Form (MHC-SF), are suitable options. However, for a more focused evaluation of the central construct, the Wellbeing Conceptual Framework (WBCF) offers the most accurate assessment of flourishing. In all cases, these measures have demonstrated strong validity by providing a robust evaluation of the central construct. Therefore, depending on the specific focus (broad or narrow) of the practitioner or researcher, any of these questionnaires may be a suitable choice.

## Data Availability

The datasets, analytic scripts, and figures are available at https://osf.io/ybrnz/.
